# An ecological momentary assessment study of physical activity behaviors among mothers of toddlers from low-income households

**DOI:** 10.1186/s12905-021-01243-2

**Published:** 2021-03-22

**Authors:** Katherine L. Campbell, Yan Wang, Ann Pulling Kuhn, Maureen M. Black, Erin R. Hager

**Affiliations:** 1 Department of Biology, Emory University, Atlanta, GA 30322 USA; 2 Department of Pediatrics, Growth and Nutrition Division, University of Maryland School of Medicine, Baltimore, MD 21201 USA; 3 Department of Epidemiology and Public Health, University of Maryland School of Medicine, Baltimore, MD 21201 USA; 4 RTI International, Research Triangle Park, NC 27709 USA

**Keywords:** Public Health, Physical Activity, Maternal Health, Ecological Momentary, Assessment

## Abstract

**Background:**

Mothers of young children from low-income communities may be vulnerable to barriers associated with low physical activity. The purpose of this study was to examine associations between home environment factors and maternal physical activity among mothers of toddlers.

**Methods:**

Mothers of toddlers (n = 200) recruited from low-income communities simultaneously wore an ankle-placed accelerometer and were given a personal digital assistant for ecological momentary assessment. Mothers received randomly prompted questions about their current environment, activity, and social setting several times a day over eight consecutive days. Data were analyzed using linear mixed-effects regression models with random intercepts; within-group and between-group relations between physical activity and environment factors were disaggregated.

**Results:**

Within-group relations included higher physical activity counts for specific mothers with television off versus on (95% CI = 130.45, 199.17), children absent versus present (95% CI = 82.00, 3.43), engaging with a child versus not (95% CI = 52.66, 127.63), and outside versus inside location (95% CI = 277.74, 392.67). Between-group relations included higher physical activity on average when other adults were absent versus present (95% CI = − 282.63, − 46.95). Recruitment site (urban vs. semi-urban) significantly moderated the within-group relation between being outside versus inside and activity count (β = − 243.12, 95% CI = − 358.74, − 127.47), and showed stronger relations among urban mothers (β = 440.33, 95% CI = 358.41, 522.25), than semi-urban (β = 190.37, 95% CI = 109.64, 271.11). Maternal body weight significantly moderated the within-group relation between being located outside versus inside the home and activity count (β for interaction = − 188.67, 95% CI = − 308.95, − 68.39), with a stronger relation among mothers with normal weight (β = 451.62, 95% CI = 345.51, 557.73), than mothers with overweight/obesity (β = 271.95, 95% CI = 204.26, 339.64).

**Conclusions:**

This study highlights home environmental factors, including screen time, the presence of others (adults and children), and location (i.e., outside versus inside) that may relate to maternal physical activity behaviors. Understanding factors associated with physical activity could reduce physical activity disparities.

*Trial registry* ClinicalTrials. NCT02615158, April 2006

## Background

Physical activity is a central component of a healthy lifestyle for reducing poor health outcomes [1]. Only about half of US adults report that they meet the nationally recommended levels of physical activity (50.2% in 2017) [2, 3]. Disparities in physical activity level by socio-demographic factors exist, such that lower physical activity is associated with low-income status, Black race or Hispanic ethnicity, and female gender [4–11]. These associations suggest that women from low-income communities may experience barriers to meeting the recommended physical activity levels [12].

Based on the Ecological Model of Active Living, an individual’s physical activity behavior is dependent on their social context and physical environment, including caregiving activities, physical location (outside vs. inside), work patterns, electronic usage, and social support [13–18]. Frequent television viewing and lack of time have been associated with low physical activity in children, whereas social support has been associated with increased physical activity in mothers of young children [17, 19]. These social and community-level factors, in combination, may be related to physical activity behaviors among mothers of young children living in low-income communities [15].

Ecological Momentary Assessment (EMA) is a recurrent, real-time assessment of an individual’s behavior that minimizes recall bias by addressing the context in which these behaviors are occurring [20]. A recent EMA study found higher toddler physical activity when outside (versus inside; with stronger associations observed for older children versus younger), other children were nearby (versus no other children present), and engaging with the mother (versus no engagement) [21]. This manuscript extends the toddler study by examining the association between the home environment and physical activity among mothers of toddlers using the same constructs. To expand the knowledge-base on mother and toddler physical activity, we tested the hypotheses that mothers are more active when: (1) outside versus inside, (2) TV off versus on, (3) other adults present versus absent, 4) children present versus absent, and (5) engaging with their toddler versus not engaging. Further exploratory analysis examined whether recruitment site (urban versus semi-urban) and Body Mass Index (BMI) moderated relations between environment factors and physical activity.

## Methods

### Study sample

Baseline data from the Toddler Overweight Prevention Study (TOPS), collected from 2007 to 2010, were used for this study [22]. TOPS is a randomized controlled trial that tested the effects of maternal lifestyle or responsive parenting interventions on reducing the rate of BMI increase among toddlers and mothers from urban and semi-urban areas in a Mid-Atlantic USA state [22].

We recruited samples from two sites: (1) an urban site (city of > 250,000) from a primary care clinic serving a low-income community and (2) an adjacent semi-urban site (city of < 100,000) from a WIC Clinic (Supplemental Nutrition Program for Women, Infants, and Children), a federal program that provides supplemental food, nutrition counseling, and referrals to women, infants, and children up to age five at nutritional risk and living below 185% of the federal poverty level [22, 23]. Recruitment strategies included promotional flyers and in-person recruitment in clinic waiting rooms. Baseline and follow-up data collection took place in a laboratory setting and in the home and the interventions were conducted in community sites. Eligibility criteria for toddlers included: toddler’s age 12–32 months, born at term with birth weight over 2500 g, without health restrictions, congenital problems or developmental delays, and able to walk independently. Eligible criteria for mothers included: age 18 years or older, not pregnant, WIC-eligible, English-speaking, and without medical/physical conditions that limited physical activity (Physical Activity Readiness Questionnaire, PAR-Q) [24]. The TOPS study methods are described elsewhere [25]. The University of Maryland, Baltimore Institutional Review Board (HP-00040253), and Maryland State Department of Health Institutional Review Board (Protocol 06-53) approved TOPS, including all activities and procedures in this study. The mothers provided written informed consent.

## Measures

### Sample characteristics

At baseline, mothers were electronically surveyed on demographics and health history for themselves and their toddlers [22]. In the demographic survey, mothers reported their age, toddler age, number of children, race, marital status (i.e., unmarried, married, divorced), level of education, employment status (i.e., employed/part-time or unemployed), and annual household income. To calculate mothers’ body mass index (BMI), data collectors weighed mothers (kilograms) using the TANITA 300GS (Tanita Corp) in duplicate or triplicate to the nearest 0.1 kg and measured their height in duplicate or triplicate to the nearest 0.5 cm using a Shorr measuring board (Shorr Productions). BMI was categorized as underweight (BMI < 18.5 kg/m^2^), normal weight (BMI = 18.5 to < 25 kg/m^2^), overweight (BMI ≥ 25 to < 30 kg/m^2^), and obese (BMI ≥ 30 kg/m^2^).

### Ecological momentary assessment

Home environment factors were assessed with EMA methodology. The study participants were given a personal digital assistant (PDA), PalmZ22 (Palm, Inc., Sunnyvale, CA, USA), which randomly beeped on 53 separate occasions (8:30am to 8:30 pm) over eight consecutive days to prompt participants to answer questions about their current environment, activity, and social setting [21]. The EMA methods developed for this study were pilot-tested with a small group (n = 10) of mothers with toddler-aged children to determine acceptability of question wording, optimal number of prompts per day, and timing of first and last prompt. Modifications were incorporated into the final protocol. When prompted via PDA, participants had 15 min to respond to the questionnaire. Participants were asked about engagement with their child only if they responded that the child was nearby (i.e. are you talking, singing, or playing with your child right now?). Participants who responded “no” to child nearby were not included in the analyses for engaging with child. Maternal response rate was calculated based on the number of EMA prompts answered out of the total possible prompts sent over the study period.

To account for the recurrent EMA prompts, person-mean centering was used to find the average response for each participant and the variability of their responses. Centering involved creating a mean for each participant (average of all responses for a single construct) and measuring the deviation from each participant’s personal mean for each specific response. Within-group findings represent the relationship between home environment and maternal physical activity for individual participants (i.e. the variability of physical activity of a participant, dependent on the home environment factors). Between-group findings represent the relationship between the home environment and physical activity across all participants (i.e. the average physical activity count for each participants’ completed responses, in relation to their average reported environment factors).

### Accelerometry

Physical activity was assessed using accelerometry [21]. We attached an Actical accelerometer (Phillip Respironics, Bend, OR, USA) to the non-dominant or left ankle using a non-removable hospital band placed next to the skin of the study participants [21]. The Actical accelerometer is small, waterproof, and measures movement in multiple planes. Accelerometers were worn for > 8 days for a 24-h time period to track the mothers’ and toddlers’ physical activity [21]. Raw activity counts collected in 1-min epochs were used in the analysis. If a valid EMA response was recorded, accelerometry data were summed for 15-min preceding and following the response (31 min in total). The outcome variable was the total accelerometer activity count surrounding the corresponding EMA responses (dependent variable). The dependent variable, activity counts over 31 min surrounding each beep (± 15 min), was skewed (skewness = 3.59); we conducted log transformation to normalize the data. The log transformed and non-transformed outcomes were examined, and the findings matched regarding significance and direction; non-transformed raw activity counts were used in the analysis to facilitate interpretation. To describe the amount of time mothers spent in moderate to vigorous physical activity (MVPA) per day, the threshold of > 3200 counts/minute was applied to the entire data collection period (> 8 days) [26]. The use of Actical ankle accelerometry has been found to be a valid and reliable measure of physical activity [26].

## Statistical analysis

Chi-square tests for categorical variables and t-tests for continuous variables were used to assess differences in baseline characteristics and response rate between individuals included and excluded from the analysis. Response rate by prompt time (e.g. morning versus evening, weekday versus weekend) was also assessed.

Linear mixed-effects regression models with random intercepts were used to assess the real-time relation between activity counts collected using accelerometry and environmental factors based on EMA responses, accounting for the clustering of responses within each mother. Separate models assessed activity counts in relation to environmental factors (TV on/off, presence of adults, presence of children, engagement with child, physical location) based on EMA responses, respectively. Recruitment site, number of children, time of day, age, and maternal BMI were included in the models as covariates [27]. Race/ethnicity was highly correlated with recruitment site and therefore was excluded as a covariate to avoid collinearity (non-Hispanic White/other compared to urban/semi-urban r = 0.63; p < 0.001).

We explored the moderating effects of recruitment site and maternal BMI on the relations between each home environment factor and activity counts, by including the interaction terms in the models. The moderating effect of engaging (i.e. talking, singing, playing) with the child was also explored using similar models with interaction terms for between-group and within-group relations, separately. Stratified analyses by recruitment site and by maternal weight followed if significant interactions existed (Table 4). SPSS (IBM SPSS Statistics 22) and SAS 9.4 statistical software (SAS Institute Inc, Cary, NC, USA) were used for the analyses.

## Results

### Sample characteristics

The analysis sample included 200/285 mothers (inclusion rate = 70%) with matched EMA and physical activity data. Reasons for missing data were: incomplete or missing accelerometry data (n = 48), incomplete or missing EMA data (n = 24), or EMA/accelerometry data did not overlap (n = 13).

The sample is described in Table 1. The median age was 26.2 years old and the majority of mothers identified as Black (62%). Many of the mothers were living at or below the federal poverty ratio (67%), unmarried (66%), having overweight/obesity (72%), and had two or more children in the household (median of 2.0 children). The median daily MVPA was 20.2 min. Compared to included participants, excluded participants were more likely to reside in the semi-urban versus urban site (45.5% vs 27.9%, p < 0.001) and less likely to be Black versus white (62.0% vs 80.5%, p < 0.001).

**Table 1:** Sample description (N = 200)

Median (IQR) or N (%)		
Maternal age	Years	26.2 (7.7)
Toddler age	Months	19.8 (9.5)
Household composition	Number of children	2.0 (2.0)
Physical Activity	Activity counts (counts min ^−1^)	291.2 (138.1)
	MVPA (minutes/day)	20.2 (19.1)
Maternal race	Non-Hispanic Black or African-American	124 (62.0%)
	Hispanic or Latino	7 (3.5%)
	Non-Hispanic White or Caucasian	62 (31.0%)
	Other	7 (3.5%)
Maternal body size	Underweight (BMI^a^ < 18.5)	2 (1.5%)
	Healthy weight (18.5 ≤ BMI < 25)	54 (26.9%)
	Overweight (25 ≤ BMI < 30)	40 (20.3%)
	Obese (BMI ≥ 30)	101 (51.3%)
Marital status	Unmarried	132 (66.0%)
	Married	59 (29.5%)
	Divorced	9 (4.5%)
Education	Less than high school	34 (17.0%)
	High school diploma/equivalent	69 (34.5%)
	Some college	68 (34.0%)
	College or graduate degree	29 (14.5%)
Employment	Employed/Part-time	72 (36.0%)
	Unemployed	128 (64.0%)
Socioeconomic status	Living above federal poverty ratio	65 (32.5%)
	Living at/below federal poverty ratio	130 (65.0%)
Recruitment location	Semi-urban	91 (45.5%)
	Urban	109 (54.5%)
Toddler age	≤ 24 months	147 (73.5%)
	> 24 months	52 (26.0%)

^a^BMI (body mass index)

### Ecological momentary assessment (EMA)

Among the 200 participants with matched EMA and accelerometer data, 3082 individual EMA responses were recorded that aligned with surrounding accelerometer data and comprised the analysis sample data (see Table 2). Using a denominator of 10,600 possible beeps (200 participants × 53 maximum possible prompts), EMA response rate for the study population was 39% (4106 answered prompts /10,600 possible prompts sent). Maternal response rate for the sample ranged from 4 to 85%. The mean number of responses per participant was 21, ranging from 2 to 45 responses. The distribution of responses across time of day includes 26.2% of responses in the morning (0830–1159), 28.8% in the afternoon (1200–1559) and 45.1% in the evening (1600–2030). There was no significant difference in response rates between participants with or without accelerometer data. Participants were asked about engagement with their child only if they responded that the child was nearby (i.e. are you talking, singing, or playing with your child right now?). Participants who responded “no” to child nearby were deleted from the analyses for engaging with child.

**Table 2:** Ecological momentary assessment responses (Responses = 3082; N = 200)

Factor	Question	Response Options	Variable of Interest
Television On/Off	Is the TV on in the area?	No; yes	41.4% (yes)
Adults Present	How many adults are in the room/area?	0; 1 or more	59.9% (1 or more)
Children Present	How many children are in the room/area?	0; 1 or more	72.0% (1 or more)
Interaction with Child	Are you talking, singing, or playing with your child right now?	No; yes	57.3% (yes)
Physical Location	Are you…	Inside; outside	8.5% (outside)

### Physical activity and environment

Within-group analyses showed significance for four environment factors in relation to physical activity over 30 min (see Table 3). TV off versus on was associated with 165 more counts of physical activity (95% CI = 130.45, 199.17). Not having a child nearby versus having a child nearby was associated with 43 more counts of physical activity (95% CI = 82.00, 3.43). Engaging with a child versus no engagement was associated with 90 more counts of physical activity (95% CI = 52.66, 127.63). Outside versus inside location was associated with 335 more counts of physical activity (95% CI = 277.74, 392.67). The only hypothesized factor not associated with physical activity in within-group analyses was the presence of other adults nearby.

**Table 3:** Associations between physical activity and environment factor using unadjusted and adjusted linear mixed-effects regression models

	Unadjusted Model^a^				Adjusted Model^ab^			
	Between subject		Within subject		Between subject		Within subject	
	β (95% CI)	*p*	β (95% CI)	*p*	β (95% CI)	*p*	β (95% CI)	*p*
Television: off	146.41 (21.35, 271.47)	**0.022**	164.60 (130.25, 198.94)	** < .001**	121.02 (− 6.83, 248.88)	0.063	164.81 (130.45, 199.17)	** < .001**
Adults nearby: no	151.51 (31.24, 271.78,)	**0.014**	− 9.26 (− 44.92, 26.41)	0.611	164.79 (46.95, 282.63)	**0.006**	− 11.34 (47.31, 24.62)	0.536
Children nearby: no	40.54 (− 90.62, 171.70)	0.543	45.21 (6.06, 84.35)	**0.024**	41.50 (− 87.61, 170.62)	0.527	42.71 (3.43, 82.00)	**0.033**
Engaging with child: yes	− 25.68 (− 189.30, 137.94)	0.757	88.23 (50.79, 125.67.39)	** < .001**	− 13.77 (− 180.45, 152.91)	0.871	90.15 (52.66, 127.63)	** < .001**
Physical Location: Outside	202.42 (− 105.64, 510.48)	0.197	333.89 (276.52, 391.26)	** < .001**	258.48 (− 45.50, 562.47)	0.095	335.21 (277.74, 392.67)	** < .001**

Note: Boldface indicates statistical significance (p < 0.05)

^a^Non-normalized activity counts are used in both models due to similarity to log-transformed activity counts

^b^Model adjusted for maternal age, time of day, maternal BMI, recruitment location, and number of children

Between-group analyses were significant for a single factor (see Table 3). The accompaniment of other adults versus no other adults was associated with 165 fewer counts of physical activity over 30 min on average (95% CI = − 282.63, − 46.95). Hypothesized factors not associated with physical activity in between-group analyses include TV on versus off, being near a child versus not being near a child, engaging with a child versus not engaging with a child (if a child is reported to be nearby), or outside versus inside location.

### Moderating effects

Recruitment site significantly moderated the within-group relation between being outside versus inside and activity count (β = − 243.12 for interaction, 95% CI = − 358.74, − 127.47) (see Table 4). Based on stratified analysis, relations between outside location and activity count were stronger among urban mothers (β = 440.33, 95% CI = 358.41, 522.25), than among semi-urban mothers (β = 190.37, 95% CI = 109.64, 271.11). Across both sites, mothers’ activity counts were higher when they were outside versus inside.

**Table 4:** Associations between physical activity and environment factor using interaction terms in linear mixed-effects regression models

Interaction model^a^	Between subject		Within subject	
	β (95% CI)	*p*	β (95% CI)	*p*
Physical Location by Recruitment Location	− 73.48 (− 687.66, 540.69)	0.814	243.12 (127.47, 358.74)	** < .001**
Semi-Urban	*179.20* *(*− *279.21, 637.61)*	*0.777*	190.37 (109.64, 271.11)	** < .001**
Urban	*324.99* *(*− *104.30, 754.28)*	*0.136*	440.33 (358.41, 522.25)	** < .001**
Physical Location by BMI	29.86 (− 694.77, 754.50)	0.935	188.67 (68.39, 308.95)	**0.002**
Normal	*314.28* *(*− *187.76, 816.31)*	*0.214*	451.62 (345.51, 557.73)	** < .001**
Overweight/Obese	*243.63* *(*− *131.53, 618.80)*	*0.201*	271.95 (204.26, 339.64)	** < .001**
Engaging with Child by BMI	*206.67* (− 148.45, 561.80)	*0.252*	− *21.68* (− 103.37, 60.00)	*0.603*
Normal	− *201.10* *(*− *438.71, 36.50)*	*0.095*	*104.47* *(33.05, 175.89)*	*0.004*
Overweight/Obese	*54.79* *(*− *171.92, 281.51)*	*0.633*	*84.42* *(40.21, 128.63)*	< *.001*

Note: Boldface indicates statistical significance (p < 0.05)

^a^Non-normalized activity counts are used in both models due to similarity to log-transformed activity

Maternal body weight significantly moderated the within-group relation between outside versus inside and activity count (β for interaction = − 188.67, 95% CI = − 308.95, − 68.39). A stratified analysis by maternal weight status found that the relation between outside location and activity counts was stronger among mothers who had normal weight (β = 451.62, 95% CI = 345.51, 557.73), than among mothers who had overweight/obesity (β = 271.95, 95% CI = 204.26, 339.64).

There were no significant between-group moderating effects of recruitment site and maternal BMI for physical location (inside vs. outside). Maternal weight status did not moderate the within or between-group relation between engaging with a child versus not engaging and activity count. Furthermore, there was not a moderating effect for response rate.

## Discussion

Using EMA in conjunction with accelerometry in real time, five factors within the Ecological Model of Active Living were tested, yielding within-group (for individual participants) and between-group (average of all participants) findings. Study findings align with the guiding model by identifying home environment factors associated with maternal physical activity such as screen time, the presence of others (i.e., adults and children), physical location (i.e., outside vs. inside) as well as moderating factors such as maternal weight status and recruitment site (i.e., urbanicity).

The within-group finding that individual participants were less active when the television was on (versus off) extends similar findings that television usage is associated with low physical activity, as reported on recall surveys [15, 28]. In contrast, a study using the same dataset found no relation for toddler physical activity between television on versus off [21]. We did not collect data on the type of television programming and whether it was geared towards mothers or toddlers. Future research should explore the type of programming and the intended audience (mother vs. toddler) and how this may affect their physical activity levels.

Findings related to physical activity in the context of other adults varied. Across participants, mothers who reported being alone more frequently had more physical activity counts over 30 min compared to mothers who reported being with other adults. However, among individual mothers, rates of physical activity were not associated with the presence of other adults, possibly due to competing contexts of why other adults were present. For example, mothers may be with other adults at a sedentary job or with other adults who are providing social support to be physically active, as shown in other studies [18]. Future studies may prioritize understanding the context of the social setting to parse out associations with physical activity.

Contrary to expectations, individual mothers had lower activity counts when in the presence of one or more children compared to when they were alone. Previous studies have shown that maternal and child physical activity are associated, including a similar study on toddlers in this same population [21]. Toddlers often engage in “secure base” behaviors, in which they play in the vicinity of their caregivers, running back and forth to check-in [29], which does not require much activity for mothers to supervise their child [30]. Although when individual mothers were engaging with their child, they were more active compared to when they were together, but not engaging with their child. In the toddler study, the toddlers were also more active when engaging with the mother, supporting the connection between mother and toddler physical activity [21]. These findings suggest that mothers are most active without their toddler nearby. Future studies could examine whether mother physical activity drives toddler physical activity or vice versa, since mothers in low-income communities are exposed to barriers associated with low physical activity [12].

The finding that individual mothers and mothers on average (marginally) were more active when outside (versus inside) is consistent with the toddler study, where within individual toddlers, physical activity counts were higher when outside compared to inside [21]. Outside location could be associated with exercise or active transportation if there are yards, sidewalks, or public transit near the home that provide opportunities to be physically active [15]. In this study, the availability of green space and perhaps the accessibility and infrastructure of surrounding areas, including parks and walking paths, may encourage physical activity and active transport when outdoors [15, 31].

Recruitment site significantly moderated the within-group relation between physical location and physical activity among participants. Mothers recruited from the urban site had higher activity counts when outside compared to inside, which was greater than the activity counts of mothers recruited from the semi-urban site. Prior studies have found that active transport is associated with physical activity, which may be more prevalent in the urban sample compared to the semi-urban sample [15]. Social environmental factors that may restrict physical activity, such as crime, poverty, and maintenance of infrastructure, may impact both the urban and semi-urban mothers [32]. Decreased access to exercise facilities may also support these findings because mothers may be getting their activity mainly outdoors from transport, instead of exercising in a facility or indoors, as they are a relatively sedentary group [33]. Previous literature suggests that individuals of low socioeconomic status often get their physical activity from physically demanding occupations, which may apply in this sample [34].

This study found a difference in physical activity by weight status among mothers when outside versus inside such that mothers with overweight/obesity were less active when outside compared to mothers who had normal weight status. In general, adult healthy weight women are more active compared to women with overweight/obesity [27, 35]. These findings suggest that targeting active transport and more time outside of the home among mothers with overweight/obesity and their toddler age children may be an area for future intervention.

## Limitations

There are limitations that should be considered. First, the direction of the relations between home environmental factors and physical activity cannot be determined in this repeated cross-sectional investigation due to the contemporary relations (15 min of physical activity before/after prompt). Second, the mean response rate is low for EMA responses among mothers, with a wide range of response numbers per participant. However, this rate is an estimate using the maximum possible prompts available for each person as the denominator. We are unable to know if the non-responses were due to true participant non-response or due to device-related reasons, (e.g., non-functional battery) or if the device was returned before all 53 prompts reached the participant. These factors can be taken into consideration when calculating the response rate for future studies. Despite the limitations, there was not a moderating effect of participant response rate. Furthermore, the EMA questions were designed to be brief and did not require the participant to report the details of the context, only whether they were or were not in a particular setting. For example, the question asked whether the TV was on, not whether the mother or toddler were watching. Additionally, questions asked about the presence of other adults or children and whether mothers were engaged with the child, but they did not ask for information around the specifics of the activities. Given the limited number of EMA studies conducted with low-income women to understand the context of their physical activity, it is difficult to compare the current results with other studies. Therefore, future research is needed to compare how context relates to physical activity in other populations and settings. By combining other environmental measures, such as Geographic Information Systems (GIS) with EMA and accelerometry, we could gain a greater understanding of the home and community environmental context, including outdoor space, which was not examined in the current study.

## Conclusions

Guided by the Ecological Model of Active Living and through a novel approach using EMA paired with accelerometry, this study identified home physical and social environment factors that were associated with maternal physical activity. To increase maternal physical activity, findings call for a reduction in screen time, increasing active mother-toddler engagement in play, and promoting active transport methods. Additionally, findings suggest that future interventions target mothers with overweight/obesity and from semi-urban areas as they may be particularly vulnerable to low physical activity. Understanding factors associated with physical activity during this critical period in a woman’s life could help reduce disparities surrounding inactivity.

## Data Availability

The datasets used and/or analyzed during the current study are available from the corresponding author upon reasonable request.

## Abbreviations

BMI: Body mass index
EMA: Ecological Momentary Assessment
MVPA: Moderate to vigorous physical activity
PDA: Personal digital assistant
SEM: Social Ecological Model
TOPS: Toddler Overweight Prevention Study (a randomized controlled trial that tested the effects of maternal lifestyle or responsive parenting interventions on reducing the rate of BMI growth among toddlers and mothers
WIC: Supplemental Nutrition Program for Women, Infants, and Children
